# Effect of Concomitant Tricuspid Valve Repair on Clinical and Echocardiographic Outcomes in Patients Undergoing Left Ventricular Assist Device Implantation

**DOI:** 10.3390/jcm14217554

**Published:** 2025-10-24

**Authors:** Olga N. Kislitsina, Sandeep N. Bharadwaj, Tingqing Wu, Rebecca Harap, Jane Kruse, Esther B. Vorovich, Jane E. Wilcox, Clyde W. Yancy, Patrick M. McCarthy, Duc T. Pham

**Affiliations:** 1 Department of Medicine, Division of Cardiology, Feinberg School of Medicine, Northwestern University, Chicago, IL 60611, USA; tingqing.wu@nm.org (T.W.); rebecca.wetzel@nm.org (R.H.); esther.vorovich@nm.org (E.B.V.); jane.wilcox@nm.org (J.E.W.); cyancy@nm.org (C.W.Y.); 2 Department of Surgery, Division of Cardiac Surgery, Feinberg School of Medicine, Northwestern University, Chicago, IL 60611, USA; sandeep.bharadwaj@nm.org (S.N.B.); jkruse@nm.org (J.K.); patrick.mccarthy@nm.org (P.M.M.); dpham1@nm.org (D.T.P.)

**Keywords:** left ventricular assist device, LVAD, tricuspid regurgitation, strain, right ventricular failure

## Abstract

**Objectives:** The purpose of this study was to determine whether concomitant tricuspid valve repair (TVr) at the time of left ventricular assist device (LVAD) implantation improves outcomes in patients with ≥moderate tricuspid regurgitation (TR) and to evaluate the prognostic value of preoperative right ventricular (RV) strain. **Methods:** In a retrospective analysis of 100 LVAD recipients (44 TVr; 56 No-TVr), preoperative (preop) and postoperative (postop) clinical, echocardiographic, and hemodynamic variables, including pulmonary vascular resistance (PVR) and pulmonary artery pulsatility index (PAPI), were analyzed. RV free wall strain (RV-FWS) and RV fractional area change (RV-FAC) were measured by speckle tracking. Early right heart failure (RHF) was modeled with multivariable logistic regression, and 2-year mortality was assessed with Fine–Gray competing risk regression. Preoperative and three-month measurements were compared within each of the 100 patients. **Results:** Baseline invasive hemodynamics, RV-FWS, and RV-FAC were similar between the TVr and No-TVr groups. TVr at the time of LVAD implantation reduced postoperative TR grade, but it did not improve RV-FWS or RV-FAC at 3 months. The No-TVr patients were more often discharged home and had lower 30-day readmissions. PVR was comparable preoperatively and at 3 months postoperatively. In adjusted analyses, preop PVR, PAPI, and TVr were not independently associated with early RHF, whereas decreased preoperative RV-FWS and lower preop RV-FAC independently predicted higher 2-year mortality. **Conclusions:** In LVAD recipients with ≥moderate TR, concomitant TVr lowers postoperative TR severity but does not improve early RHF, RV strain-based remodeling, or 2-year mortality. Preoperative RV deformation metrics, rather than preoperative PVR or PAPI, independently predict survival following LVAD implantation with or without TVr.

## 1. Introduction

Functional tricuspid regurgitation (TR) typically reflects right-sided remodeling rather than primary leaflet disease. Progressive right atrial and right ventricular dilation alters tricuspid annular geometry and sub-valvular tethering, producing abnormal leaflet coaptation and a cycle of increasing tricuspid regurgitation, volume overload, and adverse remodeling. In advanced heart failure, these mechanisms are amplified by elevated right-sided filling pressures, pulmonary vascular load, and atrial fibrillation [1].

As many as one-half of all patients undergoing LVAD support suffer from moderate or greater TR [2]. LVAD implantation unloads the left ventricle and can reduce functional MR and may also reduce TR in some patients. Despite improvements in right heart hemodynamics and TR following LVAD implantation, preoperative TR has been associated with the development of postoperative right heart failure (RHF), necessitating prolonged inotropic support and hospital stays following LVAD implantation [2,3,4,5,6]. However, LV unloading by the LVAD does not necessarily reverse intrinsic RV myopathy, nor does it address leaflet tethering or RV afterload sensitivity early after surgery. This study evaluates whether TVr confers advantages in early postoperative RHF, RV mechanics, or survival during LVAD implantation and examines the potential prognostic role of preoperative RV deformation [7].

Residual post-LVAD TR has also been associated with increases in morbidity and mortality [8]. The ability to predict post-LVAD RHF is difficult. Speckle-tracking echocardiographic strain measurements offer a non-invasive way of accurately tracking ventricular function over time, and they can facilitate the early detection and treatment of RHF. However, the preoperative strain in predicting postoperative outcomes following LVAD implantation remains unclear.

We tested whether concomitant tricuspid valve repair (TVr) at the time of LVAD implantation improves outcomes in patients with preoperative ≥ moderate TR. We hypothesized that by reducing residual post-LVAD TR, TVr would reduce early postoperative RHF, enhance postoperative right ventricular (RV) remodeling, and lower 2-year mortality. We also suspected that preoperative RV free wall longitudinal strain (RV-FWS) and RV fraction area change (RV-FAC) would be capable of predicting early RHF and 2-year mortality. We also evaluated whether preoperative pulmonary vascular resistance (PVR) and pulmonary artery pulsatility index (PAPI) have any impact on postoperative outcomes.

## 2. Patients and Methods

### 2.1. Ethical Statement

The prospective Bluhm Cardiovascular Institute database was retrospectively queried to identify all consecutive patients at our institution undergoing initial LVAD implantation with moderate or greater TR between May 2008 and December 2017. LVAD models used in this cohort were HeartMate II, HeartMate 3 (Abbott, Chicago, IL, USA) and HVAD (Medtronic, Dublin, Ireland). This database has been approved for research by the Northwestern Institutional Review Board (IRB # STU0012288) and includes consent for publication.

### 2.2. Study Design

Patients who received an RVAD or BiVAD were excluded. Of the 100 patients who met inclusion criteria, 56 received concomitant TVr with LVAD implantation (TVr group), and 44 did not receive concomitant TVr (No-TVr group). The decision to perform TVr was made by a multidisciplinary care team consensus and a tricuspid annulus size criterion of >4 cm.

Preoperative and postoperative clinical status, invasive hemodynamic measurements, and standard echocardiographic (echo) parameters were abstracted from computerized medical records. Speckle-tracking strain analysis of the echoes was performed retrospectively using TOMTEC-Arena (TOMTEC Imaging Systems GmbH, Munich, Germany) and Philips Ultrasound Workspace (Philips Healthcare, Andover, MA, USA). Imaging and hemodynamics were analyzed preoperatively, pre-discharge, at 3 months, and at 1 year following surgery. Mortality was evaluated across 2 years (with transplantation treated as a competing event).

### 2.3. Echocardiography

Tricuspid regurgitation (TR) severity was abstracted from clinical echocardiography reports and entered using a four-tier qualitative scale consistent with our lab workflow and INTERMACS capture during the study period: 0 = none/trace, 1 = mild, 2 = moderate, and 3 = severe. When reports used mixed descriptors (e.g., mild–moderate, moderate–severe), the more severe category was recorded by convention [9]. The LVOT and RVOT cardiac indices were derived from Doppler measurements as cross-sectional area × velocity-time integral × heart rate divided by body surface area. Outflow tract area was measured at the annulus and VTI by pulsed-wave Doppler per ASE guidance. Right ventricular (RV) dilatation was graded (none/mild/moderate/moderate to severe/severe) using basal and mid-cavity dimensions with qualitative assessment. Reduced RV systolic function was defined as an RV fractional area change (RV-FAC) of <35% [9].

### 2.4. Strain Analysis

**Figure 1:**
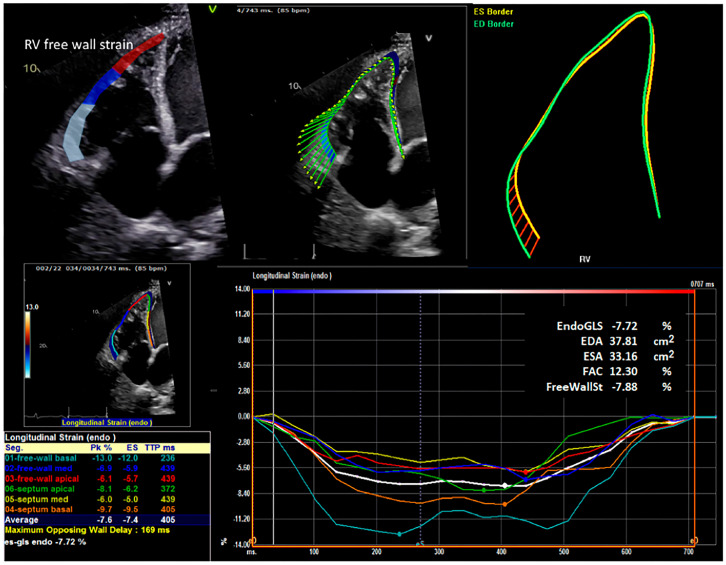
Representative image demonstrating quantifications of RV segmental wall motion, free wall strain, and RV dysfunction. The upper panels are examples of RV free wall longitudinal strain analysis in patients with advanced heart failure. The vector analysis (upper middle panel) showed reduced contraction amplitude in the distal RV free wall. The upper right panel documents the non-physiologic, rounded shape of the RV resulting from chronic pressure overload. The lower left panels are the segmental wall motion of the RV septum and the RV free wall. The lower right panel shows RV dysfunction, which is reflected by the significant reduction in peak strain derived from the time curves. The color codes are keyed to the left lower panel of segmental strains. EndoGLS = Global Longitudinal Strain; EDA = End-Diastolic Area; ESA = End-Systolic Area; FAC = fractional area change; and FreeWallSt = free wall strain.

Two-dimensional speckle tracking was performed from an RV-focused apical four-chamber view. The RV free wall longitudinal strain (RV-FWS) was defined as the mean peak systolic longitudinal strain of the basal, mid-wall, and apical free wall segments (three-segment average). The global RV longitudinal strain (RV-GLS) was defined as the mean of all six segments (three RV free wall + three RV septal segments; Figure 1).

RV strain values are reported as negative percentages, with less-negative values indicating worse deformation [10,11]. RV-FWS was prespecified as the primary strain metric; RV-GLS is reported when available for completeness. For longitudinal analyses, “improved RV dilation severity” was defined as ≥1 category reduction relative to the prior examination, whereas “postoperative RV systolic function worsening” and “postoperative TR worsening” were defined as any deterioration in their respective category grades from pre-discharge to follow-up. Continuous changes in RV-FWS and RV-FAC were also recorded.

### 2.5. Hemodynamic Parameters

Right heart catheterization (RHC) data were available preoperatively in 98 patients and at 3 months postoperatively in 62 patients. Pulmonary vascular resistance (PVR) was calculated as 80 × (mean pulmonary artery pressure − pulmonary capillary wedge pressure)/cardiac output and is reported in dyn·s·cm^−5^ (Wood units = dyn·s·cm^−5^/80). Pulmonary artery pulsatility index (PAPI) was calculated as (pulmonary artery systolic − pulmonary artery diastolic pressure)/right atrial pressure; higher values indicate better RV pulsatility. The use of inotropic support at the time of assessment was recorded when available.

### 2.6. Clinical Variables and Operative Details

The Ambler score was abstracted as a numeric perioperative risk index (higher values indicate greater predicted risk). Pre-discharge complications included renal failure, dialysis requirement, prolonged ventilation (>24 h), pneumonia, cardiac arrest, and stroke; renal failure followed institutional Society of Thoracic Surgeons concordant criteria, and dialysis requirement was recorded separately. Operative timing was categorized as emergent (within 24 h of presentation) or urgent (during the index hospitalization prior to elective scheduling). LVAD implantation was performed via median sternotomy with apical inflow cannulation oriented toward the mitral valve and outflow graft anastomosed to the ascending aorta on cardiopulmonary bypass. Tricuspid valve repair, when undertaken, consisted predominantly of ring annuloplasty at the surgeon’s discretion as part of institutional heart team practice. Device selection and ring type were not protocolized and are reported descriptively.

### 2.7. Endpoints

Primary clinical endpoints were early RHF, operative and perioperative mortality, postoperative complications, and 2-year mortality with transplantation modeled as a competing event. Primary echocardiographic endpoints were changes in RV size, RV-FAC, and residual TR severity. RV-FWS was prespecified as a secondary marker to evaluate associations with early RHF and 2-year mortality.

Early RHF was defined in accordance with contemporary INTERMACS and MCS-ARC-concordant criteria as any of the following within 30 days of LVAD implantation: need for temporary or durable right ventricular mechanical support; continuous inotropic support for ≥14 days after implant or inability to wean from inotropes by 48 h accompanied by clinical/hemodynamic evidence of right-sided congestion (elevated right-sided filling pressures, rising bilirubin/creatinine, or low pulmonary artery pulsatility index); or persistent signs of systemic venous congestion requiring intensification of therapy. Late RHF was defined as an unplanned hospitalization for RHF requiring intravenous (IV) diuretics and/or inotropes after the index discharge. Therefore, prolonged inotrope use is captured by definition, and diuretic escalation alone does not meet the criteria unless it precipitated RHF hospitalization.

Discharge home was defined as discharge to a private residence (with or without home health services). Discharges to inpatient rehabilitation, skilled nursing facilities, long-term acute care, or hospice were not considered “home.”

### 2.8. Statistical Analysis

Continuous variables were summarized using the mean with standard deviation or the median with interquartile range for continuous variables and counts with percentages for categorical variables. Comparisons between groups were evaluated using a two-sample *t*-test or a Wilcoxon rank-sum test for continuous variables and a chi-square or Fisher’s exact test for categorical variables. Freedom from ≥moderate TR was displayed using Kaplan-Meier methods and compared with the log-rank test. Mortality was evaluated through 2 years with transplantation treated as a competing event. Unadjusted comparisons used Gray’s test, and adjusted analyses used Fine-Gray subdistribution hazard regression, reported as subdistribution hazard ratios (sHRs) with 95% CIs treating transplantation as a competing event. Proportional hazards assumptions were assessed using Schoenfeld-type residuals and were found to not be violated. Early right heart failure (RHF) was modeled with multivariable logistic regression, reported as adjusted odds ratios (ORs) with 95% CIs. To assess remodeling, we fit linear regression models for change from preoperative to approximately 3-month values (ΔRV-FWS and ΔRV-FAC). By convention, strain values are negative, and less-negative RV-FWS reflects worse deformation, so ΔRV-FWS > 0 indicates worsening and ΔRV-FAC > 0 indicates improvement.

Prespecified covariates for multivariable models were age, sex, baseline TR grade (moderate vs. moderate to severe/severe), RV dilation severity, RV-FWS, RV-FAC, pulmonary artery pulsatility index (PAPI), and pulmonary vascular resistance (PVR). Degrees of freedom were limited a priori to reduce overfitting given the sample size. Analyses used complete cases at each time point; no imputation was performed. All tests were two-sided with α = 0.05, with no adjustment for multiplicity. Analyses were conducted in SAS 9.4 (SAS Institute, Cary, NC, USA).

## 3. Results

### 3.1. Preoperative Characteristics

The study cohort included 32 females (32%) and 68 males (68%) with an average age of 54.6 ± 14.4 years (Table 1).

As expected, patients in the TVr group presented with more advanced TR and greater comorbidity burden, whereas the No-TVr patients more commonly had moderate TR and were bridged to transplantation. Specifically, the TVr group had more moderate to severe/severe TR (59% vs. 25%, *p* < 0.001) and higher rates of diabetes, atrial fibrillation, larger left atrial size, higher BMI (*p* < 0.001), higher creatinine, and prior pacemaker implantation. The No-TVr patients were more frequent recipients of LVADs as bridge-to-transplants. For echocardiography, RV dilatation was more common in the TVr group, but preoperative RV-FWS, RV-FAC, and LVOT/RVOT cardiac index in the two groups did not differ (Table 1). Preoperative RA pressure, PAP, PCWP, cardiac index, PAPI, and PVR were similar in the two groups as well (Table 2).

### 3.2. Intraoperative Characteristics

As expected, patients undergoing TVr at the time of LVAD implantation had longer cardiopulmonary bypass times, but their aortic cross-clamp times were similar. Concomitant PFO closure was also more frequent in the TVr group, and three emergent operations were performed, all of which were in the No-TVr group, while urgent operations were common in both groups (Table 3).

### 3.3. Early Postoperative Outcomes

Operative and 30-day mortality were zero in both groups, and discharge mortality was similar. Re-operation rates, ICU time, and total length of hospital stay were also similar (Table 4). However, the No-TVr patients were more often discharged and had a lower 30-day readmission rate. During the follow-up period, thirty-four bridge-to-transplant patients and eight destination patients underwent transplantation (eleven TVr and twenty-three No-TVr, and five TVr and three No-TVr, respectively; all ≤ 20 months) (Table 4).

### 3.4. TR Trajectory and RV Structure/Function

**Figure 2:**
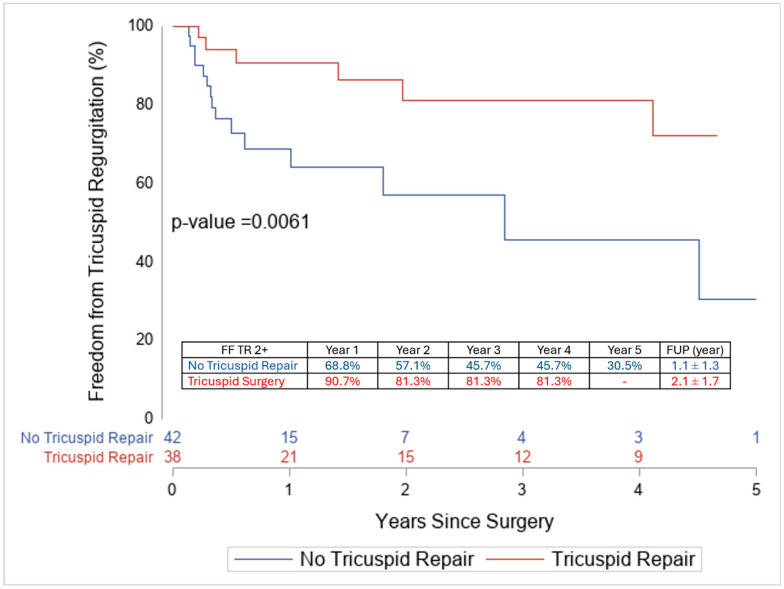
Five-year freedom from moderate or greater TR in patients with and without tricuspid valve repair at the time of LVAD implantation.

As expected, patients who received TVr at the time of LVAD implantation had experienced a marked improvement in the degree of TR by the time of discharge, and fewer of them had ≥moderate TR at 5 years (Figure 2 and Table 5).

**Figure 3:**
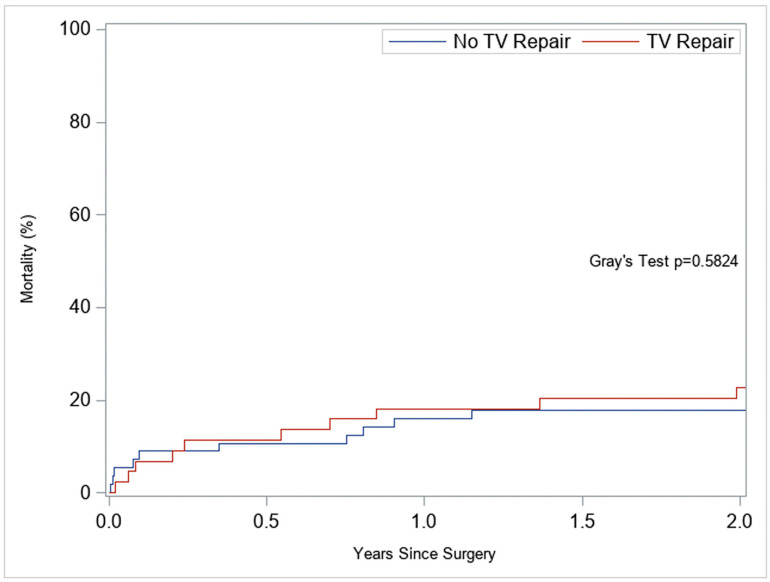
Two-year cumulative incidence of mortality after LVAD implantation in patients with (TVr) and without (No-TVr) concomitant tricuspid repair. Transplantation was modeled as a competing risk. Groups did not differ (Gray’s test *p* = 0.58).

Despite the improvement in postoperative TR, RV remodeling and deformation did not improve by approximately 3 months when TVr was performed concomitantly with LVAD implantation. The RV-FWS changed from −6.7% to −6.0%, and the RV-FAC changed from 19.6% to 17.5%. Consistent with this lack of RV remodeling following TVr, these patients had more RV dysfunction immediately post-LVAD than patients who did not require TVr (Table 5). They also showed less improvement in RV dilatation at 3 months (Table 6), and RV-FWS and RV-FAC trended towards being worse at 3 months. Predictors of early postoperative RHF, including RV-FWS and RV-FAC, are summarized in Table 5. At 2 years, the cumulative incidence of mortality (treating transplantation as a competing event) was similar between the TVr and No-TVr groups (Gray’s test *p* = 0.58; Figure 3). During follow-up, two patients required RV mechanical support (TVr 1/44 vs. No-TVr 1/56); both occurred more than 30 days after LVAD implantation.

Using multivariable logistic regression, concomitant TVr was shown to be unassociated with early RHF, even when adjusted for prolonged inotrope support. PVR and PAPI were also not independent predictors of early RHF. Using Fine-Gray competing risk regression, TVr showed a borderline association with 2-year mortality, whereas worse preop RV-FWS and lower RV-FAC were significantly associated with higher mortality (Table 6).

Following TVr, during LVAD implantation, same-patient preoperative and 3-month comparisons showed no improvement in RV deformation (strain) despite marked TR reduction (Table 5 and Table 7).

Because transplantation is common in this population, we evaluated 2-year mortality using competing risk methods.

In the competing risk model of 2-year mortality (Table 8), TVr was not independently associated with mortality. For continuous predictors, effects are per 1% point change. For RV-FWS, each 1-point less-negative value (for example, −7% to −6%) was associated with a 34% higher mortality hazard at 2 years (sHR 1.34; *p* = 0.002). A 3-point difference (−8% vs. −5%) is roughly a 2.4-fold higher hazard. For RV-FAC, each 1-point lower value was associated with a 19% higher mortality hazard (sHR 1.19; *p* = 0.003), a 5-point decrement (20% vs. 15%) is also about 2.4-fold higher. Female sex was associated with lower two-year mortality, but PVR was not a significant predictor of mortality. Proportional hazards assumptions were assessed and not violated. Unadjusted cumulative incidence curves showed no difference in patients who did or did not have a TVr at the time of LVAD implantation, despite the marked difference in their preoperative clinical status (Gray’s test *p* = 0.58; Figure 3), and the results were concordant in the Fine-Gray model (Table 8).

## 4. Discussion

The TVr and No-TVr cohorts differed at baseline in TR grade and comorbidity burden, reflecting pragmatic clinical selection for concomitant TVr. To address this, we presented the same-patient preop and postop results in the TVr cohort and performed adjusted analyses that accounted for TR severity, RV size and function, and invasive hemodynamics (including PVR and PAPI). These analyses confirm the central message of our study: while TVr reliably reduces TR grade, it does not translate into improved early RHF or survival after accounting for baseline disease severity. Conversely, preoperative RV deformation metrics (RV-FWS and RV-FAC) track prognosis more closely than PVR following LVAD implantation. Annuloplasty reduces annular area and malcoaptation, thereby lowering TR grade [2,8]. However, several mechanisms can blunt early RV functional recovery after LVAD. First, intrinsic RV myopathy and tethering (including papillary -chordal geometry and septal contributions) are not corrected by annular reduction alone [1,7,11,12]. Second, interventricular dependence under LV decompression may shift septal geometry and modify RV loading conditions [5,13,14]. Third, right-sided afterload sensitivity (pulmonary vascular load, residual PVR, and RV–pulmonary arterial coupling) can limit contractile reserve in the early postoperative period [3,15,16,17]. Finally, perioperative factors (ischemia-reperfusion, transfusion, tempo of decongestion) may temporarily worsen RV mechanics [2,8]. These considerations provide a physiologic explanation for the observed dissociation between TR reduction with TVr and the lack of improvement in RV-FWS and RV-FAC or early RHF. Consistent with this, our adjusted analyses showed no independent association between TVr and early RHF or mortality, whereas preoperative RV-FWS and RV-FAC were prognostic for 2-year outcomes.

When transplantation was treated as a competing event, survival did not differ by concomitant TVr. The adjusted Fine-Gray models likewise showed no independent association between TVr and 2-year mortality, whereas preoperative RV deformation (RV-FWS, RV-FAC) remained prognostic. Taken together, these findings suggest that baseline preop RV mechanics, not the decision to repair the tricuspid valve, drive risk after LVAD, reinforcing an individualized rather than routine approach to TVr.

The primary physiologic follow-up in this study occurred at approximately 3 months, a time window that may be too early to capture structural reverse remodeling of the RV in a subset of patients. LV reverse remodeling typically precedes RV adaptation, and persistent pulmonary vascular load or tethering may delay functional gains. Therefore, our finding that RV-FWS and RV-FAC were worse at approximately 3 months in the TVr group should be interpreted as an early snapshot in time rather than a definitive absence of later improvement. Following patients longer should clarify whether any patients experience delayed benefits from TVr that were not apparent in this study.

The optimal management of TR in patients receiving LVADs remains debated. Previous reports have shown reductions in TR severity and improvements in qualitative RV function lasting up to six months following LVAD implantation. However, residual TR has been associated with prolonged inotropic support, extended hospital stays, and increased morbidity and mortality [2,3,4,5,6]. Advanced age, large preoperative TV annulus diameter, and residual MR have all been significantly associated with residual TR and have supported the adoption of concomitant valve interventions [6,10]. Reports by Fujita and Brewer report improved equivalent or superior 2-year mortality following concomitant TVr and LVAD implantation for patients with moderate to severe TR [18,19].

Opponents of concomitant repair cite no significant differences in long-term mortality with longer cardiopulmonary bypass times, a greater need for blood products, and higher postoperative creatinine and BUN levels as reasons to avoid concomitant TVr [4,17,20]. An analysis of over 2000 patients in the Society of Thoracic Surgeons database who received TV surgical procedures during LVAD implantation demonstrated an increased risk for postoperative renal failure, dialysis (both closely associated with prolonged cardiopulmonary bypass times), re-operation, and greater transfusion requirements, highlighting the benefit of TVr in selected patients due to the presence of underlying progressive pathology. This analysis and others have supported the need for further selection criteria to determine in whom concomitant TVr can prevent early postoperative RHF [15,21,22]. Our findings reinforce the view that despite reliable TR reduction following TVr, limited early RV reverse remodeling and potentially increased operative costs may blunt the clinical benefit of TVr and thus support the selective, but not routine, use of TVr at the time of LVAD implantation.

This report is one of the largest to date that evaluates the utility of both preoperative and postoperative RV strain analyses as predictors of early RHF following LVAD implantation. Prior smaller reports have suggested the preoperative RV strain to be an independent predictor of postoperative right heart failure, with lower preoperative RV-FWS associated with reduced RV-FWS after LVAD placement [3,13]. Gumus et al. concluded that an RV-FWS ≥ −15.5% and a right ventricular stroke work index < 400 mmHg mL^−1^ m^−2^ were predictive of early RHF following LVAD implantation [16]. Preoperative strain-based predictions have been reported to have a sensitivity of 85.7% and specificity of 95.4 [23]. Work by Kato et al. suggested that combining preoperative and postoperative strain measurements increased predictive accuracy to 80.9% [14]. In our study, preoperative RV-FWS and RV-FAC were similar in patients who did and did not receive TVr, and neither independently predicted early RHF when subjected to multivariable analysis (Table 6). At approximately 3 months, deformation indices in the TVr cohort were unchanged, despite a marked reduction in TR (Table 5), limiting the value of preoperative strain for predicting early RHF in routine practice. Notably, however, abnormally low preoperative RV-FWS and RV-FAC were independently associated with increased 2-year mortality, as determined by adjusted competing risk analyses (Table 8), highlighting their prognostic role in this cohort.

### Study Limitations

This was a single-center, retrospective, non-randomized study of only 100 patients and is, therefore, subject to selection bias. Patients who underwent concomitant TV repair (TVr) had more advanced TR and greater comorbidity burden than those without repair, reflecting real-world clinical selection. We addressed these baseline differences with adjusted models, but residual confounding is still possible, and the small sample size limits model complexity and power. Details of the TVr technique were not analyzed by subgroup due to limited numbers. Agent-level dosing and duration of vasoactive therapy were not collected outside of RHF adjudication; as a result, VIS and standalone inotrope duration comparisons could not be performed. Finally, the results from this center may not be generalizable to programs with different patient selection, surgical practice, or perioperative management, and our outcomes were only analyzed for up to 2 years.

## 5. Conclusions

In this observational cohort of LVAD recipients with ≥moderate TR, concomitant TVr reduced TR grade but did not improve RV strain parameters, adjusted early clinical outcomes, or 2-year mortality. After adjustment, PVR, while clinically relevant, was not independently associated with early RHF or 2-year mortality, whereas preoperative RV-FWS and RV-FAC were independent predictors of 2-year mortality following LVAD implantation. Larger studies, preferably randomized or propensity-balanced, are needed to define when TVr yields outcome benefits beyond TR reduction.

## Figures and Tables

**Table 1:** Preoperative patient characteristics.

Variable	No Tricuspid Valve Repair (n = 56)		Tricuspid Valve Repair (n = 44)		*p*-Value
Age (years)	54.2	±12.6	55.0	±16.6	0.78
Body mass index (kg/m^2^)	25.2	±4.6	29.0	±6.5	<0.001
CHA_2_DS_2_VASc score	2.9	±1.3	3.4	±1.6	0.08
Creatinine level (mg/dL)	1.1	(0.9, 1.5)	1.5	(1.0, 2.0)	0.016
Albumin level (g/dL)	3.2	(2.7, 3.5)	3.5	(3.3, 3.8)	0.003
Gender (female)	22	(39)	10	(23)	0.08
Diabetes	10	(18)	24	(55)	<0.001
Dyslipidemia	29	(52)	27	(61)	0.34
Hypertension	25	(45)	23	(52)	0.45
Chronic lung disease	20	(36)	17	(39)	0.76
Prior stroke	5	(9)	4	(9)	0.98
Prior pacemaker	21	(38)	7	(16)	0.017
Coronary artery disease	13	(42)	13	(68)	0.07
Atrial fibrillation history	19	(34)	28	(64)	0.003
Bridge-to-transplant therapy	19	(34)	34	(81)	<0.001
Preoperative Echocardiography Characteristics					
Ejection fraction (%)	15.0	(13.0, 20.0)	15.0	(10.5, 20.0)	0.80
Left atrial size (cm)	4.4	(3.9, 5.1)	5.0	(4.2, 5.5)	0.005
Left ventricular outflow tract cardiac index (L/min/m^2^)	1.6	(1.2, 2.2)	1.6	(1.4, 2.0)	0.87
Right ventricular outflow tract peak velocity (cm/s)	51.0	(40.0, 60.0)	50.0	(41.4, 60.4)	0.69
Tricuspid regurgitation peak gradient (cm/s)	41.2	(31.3, 51.7)	34.2	(26.3, 44.1)	0.06
Right ventricular free wall strain (%)	−5.1	(−9.8, −3.9)	−6.7	(−9.0, −4.5)	0.40
Right ventricular fractional area change (%)	15.9	(11.9, 21.9)	19.6	(12.6, 24.0)	0.50
Aortic insufficiency					0.53
0 = none/trivial	33	(7)	27	(82)	
1 = mild	8	(18)	5	(15)	
2 = moderate	4	(9)	1	(3)	
Mitral insufficiency					0.30
0 = none/trivial	2	(4)	1	(2)	
1 = mild	8	(15)	5	(12)	
2 = moderate	10	(19)	16	(37)	
3 = mod/sev	3	(6)	4	(9)	
4 = severe	29	(56)	17	(40)	
Tricuspid insufficiency					0.002
2 = moderate	40	(75)	18	(41)	
3 = mod/sev	3	(6)	7	(16)	
4 = severe	10	(19)	19	(43)	
Right ventricle dilation severity					0.038
Moderate–severe/severe	4	(7)	4	(9)	
Mild–moderate/moderate	13	(23)	21	(48)	
Mild/none/normal	21	(38)	13	(30)	
Unknown/missing	18	(32)	6	(14)	
Reduced right ventricular systolic function	50	(89)	42	(95)	0.19
Left ventricular assist device placed as bridge-to-transplant	38	(68)	15	(34)	<0.001
Intermacs class					0.701
1	13	(23)	10	(23)	
2	23	(41)	20	(45)	
3	8	(14)	9	(20)	
4	4	(7)	2	(5)	
Unknown	8	(14)	3	(7)	

Values are mean ± standard deviation, *n* (%), or median (interquartile range). CHA_2_DS_2_VASc = congestive heart failure, hypertension, age ≥ 75 (doubled), diabetes, stroke (doubled), vascular disease, age 65 to 74, and sex category (female).

**Table 2:** Preoperative right heart catheterization summaries.

Variable	No Tricuspid Valve Repair (n = 56)		Tricuspid Valve Repair (n = 44)		*p*-Value
Right atrial mean pressure (mmHg)	15.5	(8.0, 19.0)	17.0	(10.0, 21.0)	0.41
Right ventricular systolic pressure (mmHg)	56.5	(47.0, 63.0)	52.0	(43.0, 63.0)	0.19
Pulmonary artery systolic pressure (mmHg)	57.0	(47.0, 66.0)	50.5	(44.5, 65.0)	0.29
Pulmonary artery diastolic pressure (mmHg)	28.0	(25.0, 34.0)	28.0	(23.0, 32.0)	0.53
Pulmonary artery mean pressure (mmHg)	41.5	(35.0, 47.0)	37.5	(32.0, 46.0)	0.10
Pulmonary capillary wedge mean pressure (mmHg)	29.5	(24.0, 37.0)	27.0	(23.0, 33.0)	0.20
Pulmonary artery pulsatility index (index value)	2.0	(1.3, 2.9)	1.6	(1.0, 2.2)	0.109
Pulmonary artery saturation (% O_2_)	57.5	(44.5, 62.0)	53.0	(47.0, 58.0)	0.23
Cardiac output (L/min)	4.3	(3.5, 5.7)	3.9	(3.5, 4.7)	0.26
Cardiac index (L/min/m^2^)	2.1	(1.8, 3.3)	2.0	(1.7, 2.3)	0.12
Systolic blood pressure (mmHg)	105.0	(97.5, 118.0)	108.0	(103.0, 121.0)	0.15
Diastolic blood pressure (mmHg)	70.0	(65.0, 78.0)	74.0	(69.0, 81.0)	0.033
Mean arterial pressure (mmHg)	84.5	(78.0, 94.0)	87.3	(82.0, 97.0)	0.08
PVR_preop, median (Q1, Q3)	184.9	(111.4, 252.1)	201.2	(134.9, 310.3)	
PVR_3mo, median (Q1, Q3)	158.8	(104.7, 233.7)	162.0	(132.1, 243.2)	
Papi_preop, median (Q1, Q3)	2.0	(1.3, 3.0)	1.6	(1.0, 2.3)	
Papi_3mo, median (Q1, Q3)	2.0	(1.6, 3.8)	1.9	(1.4, 3.1)	
RV dilation severity at preop, No. (%)					0.038
Moderate–severe/severe	4	(7%)	4	(9%)	
Mild–moderate/moderate	13	(23%)	21	(48%)	
Mild/none/normal	21	(38%)	13	(30%)	

Values are median (interquartile range).

**Table 3:** Intraoperative patient characteristics.

Variable	No Tricuspid Valve Repair (n = 56)		Tricuspid Valve Repair (n = 44)		*p*-Value
Perfusion Time (min), Median (Q1, Q3)	81.0	(58.5, 109.0)	114.5	(102.0, 130.5)	<0.001
Cross-Clamp Time (min), Median (Q1, Q3)	53.0	(27.0, 69.0)	51.0	(21.0, 53.0)	0.35
TAVR, No. (%)	0	(0%)	0	(0%)	.
Aortic Valve Surgery, No. (%)	3	(5%)	2	(5%)	0.85
Mitral Valve Surgery, No. (%)	0	(0%)	1	(2%)	0.26
Pulmonic Valve Surgery, No. (%)	0	(0%)	0	(0%)	.
Other Non-Cardiac Surgery, No. (%)	0	(0%)	1	(2%)	0.26
LVA Repair, No. (%)	0	(0%)	0	(0%)	.
VSD Repair, No. (%)	0	(0%)	0	(0%)	.
ASD (PFO), No. (%)	3	(5%)	9	(20%)	0.021
Congenital Defect Repair, No. (%)	0	(0%)	0	(0%)	.
Laser Revascularization, No. (%)	0	(0%)	0	(0%)	.
Cardiac Trauma, No. (%)	0	(0%)	0	(0%)	.
AF Ablation Surgery, No. (%)	0	(0%)	0	(0%)	.
Surgery Type, No. (%)					0.15
Elective	5	(9%)	8	(18%)	
Emergent	3	(5%)	0	(0%)	
Emergent Salvage	0	(0%)	1	(2%)	
Urgent	48	(86%)	35	(80%)	

**Table 4:** Postoperative patient characteristics.

Variable	No Tricuspid Valve Repair (n = 56)		Tricuspid Valve Repair (n = 44)		*p*-Value
Ambler score	10.8	±7.2	14.1	±10.3	0.06
Total intensive care unit hours	143.7	(95.6, 200.3)	131.9	(97.0, 311.9)	0.78
Total length of stay, days	29.0	(23.0, 41.0)	28.5	(19.5, 41.0)	0.36
Postoperative length of stay, days	20.0	(13.5, 28.5)	17.5	(13.0, 25.0)	0.24
Readmitted to the intensive care unit	14	(25)	9	(20)	0.59
Pre-discharge complications	47	(84)	35	(80)	0.57
Postop stroke > 24 h	2	(4)	0	(0)	0.21
Prolonged ventilation > 24 h	35	(63)	24	(55)	0.42
Pulmonary embolism	1	(2)	0	(0)	0.37
Pneumonia	5	(9)	7	(16)	0.29
Renal failure	5	(9)	7	(16)	0.29
Dialysis required	5	(9)	5	(11)	0.69
Cardiac arrest	1	(2)	2	(5)	0.42
Postoperative atrial fibrillation	10	(18)	6	(14)	0.57
Discharged to home	38	(75)	21	(51)	0.021
Readmission within 30 days	9	(18)	17	(44)	0.007
Operative mortality	0	(0)	0	(0)	1
Discharge mortality	5	(9)	3	(7)	0.70
30-day mortality	0	(0)	0	(0)	1
Transplant	26	(46)	16	(36)	0.311

Values are mean ± standard deviation, *n* (%), or median (interquartile range).

**Table 5:** Postoperative echocardiography characteristics.

Variable	No Tricuspid Valve Repair (N = 56)		Tricuspid Valve Repair (N = 44)		*p*-Value
Pre-Discharge					
Left ventricular outflow tract cardiac index (L/min/m^2^)	0.9	(0.6, 1.4)	1.7	(1.1, 1.9)	0.17
Right ventricular outflow tract peak velocity (cm/s)	59.4	(43.6, 81.9)	64.5	(49.3, 86.5)	0.37
Tricuspid regurgitation peak gradient (cm/s)	24.2	(18.7, 29.4)	20.1	(15.2, 23.3)	0.10
Right ventricle dilated	40	(71)	36	(82)	0.34
Right ventricle dilation severity					0.22
Moderate–severe/severe	12	(21)	6	(14)	
Mild–moderate/moderate	15	(27)	13	(30)	
Mild/none/normal	6	(11)	11	(25)	
Tricuspid regurgitation					<0.001
Moderate–severe/severe	12	(21)	1	(2)	
Mild–moderate/moderate	12	(21)	5	(11)	
Mild/none/normal	23	(41)	35	(80)	
Reduced right ventricle systolic function	44	(79)	41	(93)	0.047
Three-Month Follow-Up					
Right ventricular outflow tract peak velocity (cm/s)	57.9	(48.1, 63.4)	56.1	(47.4, 66.2)	0.89
Tricuspid regurgitation peak gradient (cm/s)	22.0	(18.7, 27.2)	20.0	(18.1, 24.8)	0.25
Right ventricular free wall strain (%)	−8.0	(−10.2, −5.1)	−6.0	(−8.6, −4.2)	0.07
Right ventricular fractional area change (%)	21.5	(16.3, 27.7)	17.5	(12.7, 24.8)	0.08
Improved right ventricular dilation severity	11	(20)	2	(5)	0.005
Reduced right ventricular systolic function	33	(59)	31	(70)	0.41
Tricuspid regurgitation worsened	6	(11)	1	(2)	0.019
One-Year Follow-Up					
Reduced right ventricular systolic function	29	(52)	27	(61)	0.34
Right ventricular systolic function worsened (compared to postoperative)	3	(5)	7	(16)	0.18
Tricuspid regurgitation worsened (compared to postoperative)	2	(4)	1	(2)	0.021

Values are n (%) or median (interquartile range).

**Table 6:** Multivariable predictors of early postoperative right heart failure (RHF).

Variable	Coefficient (Log-Odds)	OR (95%CI)		*p*-Value
Age, Tear	1.00	0.95	1.07	0.89
Female vs. Male	0.37	0.02	6.03	0.48
Concomitant TV Repaired	3.58	0.25	50.26	0.34
Right Ventricular Endocardial Global Longitudinal Strain at Preop	0.62	0.33	1.14	0.12
Right Ventricular Fractional Area Change at Preop	0.83	0.65	1.07	0.15
PVR at Preop	1.01	1.00	1.013	0.11
PAPI at Preop	0.46	0.16	1.35	0.16
RV Dilation Severity at Preop, No. (%)				Overall *p*-0.49
Mild–Moderate/Moderate	0.39	−2.93	3.70	0.82
Mild/None/Normal	−1.99	−6.08	2.11	0.34
Tricuspid Regurgitation at Preop				
Mild–Moderate/Moderate	−0.41	−2.64	1.81	0.71

**Table 7:** Predictors of improvement at 3 months.

ΔRV-FWS Model		
Predictor	β (95% CI)	*p*-value
TVr (vs. No-TVr)	+1.45 (−0.18 to +3.09)	0.08
Preop RV-FWS (per 1%)	+0.28 (0.06–0.49)	0.012
ΔRV-FAC model		
TVr (vs. No-TVr)	−2.45 (−5.84 to +0.95)	0.16
Preop RV-FAC (per 1%)	+0.35 (0.15–0.55)	<0.001

**Table 8:** Fine-Gray competing risk regression for 2-year mortality (transplant as a competing event).

Predictor	sHR (95% CI)	*p*-Value
Female	0.30 (0.10–0.85)	0.023
TVr (vs. No-TVr)	3.15 (0.96–10.33)	0.058
RV-FWS (preop)	1.34 (1.12–1.60)	0.002
RV-FAC (preop)	1.19 (1.06–1.33)	0.003
PVR (preop)	0.998 (0.994–1.002)	0.24

*Notes:* sHR = subdistribution hazard ratio; CI = confidence interval; TVr = tricuspid valve repair; RV-FWS = right ventricular free wall strain; RV-FAC = right ventricular fractional area change; PVR = pulmonary vascular resistance. Units: RV-FWS per 1% less-negative strain; RV-FAC per 1% lower; PVR per dyn·s·cm^−5^. Model additionally adjusted for baseline TR grade, RV dilation severity, age, and PAPI. Proportional hazards assumptions were tested and not violated.

## Data Availability

The original contributions presented in this study are included in the article. Further inquiries can be directed to the corresponding author(s).

## Abbreviations

ASE	American Society of Echocardiography
BIVAD	bi-ventricular assist device
FAC	fractional area change
FWS	free wall strain
LVAD	left ventricular assist device
RV	right ventricular
RVAD	right ventricular assist device
RHF	right heart failure
TR	tricuspid regurgitation
TVr	tricuspid valve repair
PVR	pulmonary vascular resistance
PFO	patent foramen ovale
sHR	subdistribution hazard ratio
